# Effects of water molecules on tribological behavior and property measurements in nano-indentation processes - a numerical analysis

**DOI:** 10.1186/1556-276X-8-389

**Published:** 2013-09-17

**Authors:** Yachao Wang, Jing Shi

**Affiliations:** 1 Department of Industrial and Manufacturing Engineering, North Dakota State University, Dept 2485, PO Box 6050, Fargo, ND 58108, USA

**Keywords:** Nano-indentation, Water molecules, Tool-material interaction, MD simulation, Tribological effect

## Abstract

Nano/micro-manufacturing under wet condition is an important consideration for various tool-based processes such as indentation, scratching, and machining. The existence of liquids adds complexity to the system, changes the tool/work interfacial condition, and affects material behaviors. For indentation, it may also affect material property measurements. However, little effort has been made to study this challenging issue at nano- or atomistic scale. In this study, we tackle this challenge by investigating nano-indentation processes submerged in water using the molecular dynamics (MD) simulation approach. Compared with dry indentation in which no water molecules are present, the existence of water molecules causes the increase of indentation force in initial penetration, but the decrease of indentation force in full penetration. It also reduces the sticking phenomenon between the work and tool atoms during indenter retraction, such that the indentation geometry can be better retained. Meanwhile, nano-indentation under wet condition exhibits the indentation size effect, while dry nano-indentation exhibits the reverse indentation size effect. The existence of water leads to higher computed hardness values at low indentation loads and a smaller value of Young's modulus. In addition, the friction along the tool/work interface is significantly reduced under wet indentation.

## Background

Along with the rapid advancement of manufacturing technologies, the size of precision parts and the thickness of thin films have been significantly reduced. To identify the mechanical properties of work materials at small scales, nano-indentation is often adopted, in which an indenter with known geometry is driven into the work material. In fact, nano-indentation can also be regarded as a fundamental manufacturing process - the tool/material interaction in this process observed provides insight for other processes such as scratching and machining. Meanwhile, in any manufacturing process, the existence of a liquid between the tool and the work material will bring tribological changes to the tool/material interaction. For instance, the immediate benefits of applying lubricants in machining processes may include reduced friction on the tool/material interface, reduced cutting forces, and longer tool life. However, at nano/atomistic-scale sizes, there has been lack of investigation on the tribological effects of a liquid in tool-based manufacturing processes. As the first step, it makes sense to develop such understanding from the nano-indentation process.

In the literature, nano-indentation has been widely applied to determine the hardness values of bulk solid or thin films [1-3]. The Oliver-Pharr method [4] and work-of-indentation method [5] are the two popular approaches to determine hardness values based on load-depth curves. In the study of Zhou and Yao [6], for instance, the Oliver-Pharr method is adopted to calculate indentation hardness directly from the load-depth curve in the indentation process of single-crystal aluminum and single-crystal silicon using an atomic force microscope (AFM). A similar AFM indentation experiment was conducted by Beegan et al. [7] on sputter-deposited copper films, in which both the Oliver-Pharr method and the work-of-indentation method are used to analyze the results. Bhushan and Koinkar [8] also applied AFM to measure the hardness of ultra-thin films with an extremely small indentation depth of 1 nm by a specially prepared diamond tip. Nevertheless, the hardness measurement at the micro/nano-level exhibits strong indentation size effect (ISE), which means that the measured hardness decreases with increasing indentation depth. Especially, crystalline materials are known to have strong indentation size effect in micro-indentation hardness test. Oliver and Pharr [4] and Nix [9] performed a series of indentation tests with varying tip radii to examine the ISE. It was discovered that hardness values can be significantly affected by the indenter radius when a spherical indenter is applied. Xue et al. [10] established a model to study the effect of tip radius on micro-indentation hardness. The results show that the effect of the indenter tip radius disappears once the contact radius exceeds one half of the indenter tip radius. Moreover, to measure the indentation modulus and hardness of copper more precisely, McElhaney et al. [11] proposed a novel method to measure the contact area by taking into account the influence of inherent pile-up and sink-in in the indentation experiment of polycrystalline copper. Similarly, Ma and Clarke [12] investigated the relationship between size effect and crystal anisotropy in hardness measurement.

The existence of a liquid in nano-indentation is believed to be able to reduce the ISE. For example, Atkinson and Shi [13] investigated the apparent variation of the hardness of iron by varying the load from 15 g to 20 kg. It is found that the hardness variation is markedly reduced by liquid lubrication. This result suggests that the ISE is actually dependent upon friction condition. A similar experiment was performed by Ren et al. [14]. The load varies from 0.125 to 1 kg in the indentation process of single-crystal MgO, but the ISE is seldom affected by the addition of a liquid for this material. Li et al. [15] studied the influence of a liquid on the friction between the micro-hardness indenter and the test material. It is claimed that the friction is the major reason for the increased hardness values under low loads and the ISE is related to the surface area-to-volume ratio. Moreover, Almond and Roebuck [16] discovered that the effect of lubrication on indentation hardness is significantly related to the indenter geometry. The existence of a liquid has little effect when the indenter's inclined angle is greater than 120°.

In this study, to investigate how the existence of a liquid affects the tool/material interaction in nano-indentation, as well indentation measurements, we adopt the technique of molecular dynamics (MD) simulation. This is an effective numerical approach for studying many intriguing issues such as material deformation, dislocation propagation, phase transformation, as well as material property evaluation. Many of these issues are beyond the capability of experimental approaches under very small scales. It should be noted that MD simulation has been widely adopted in studying various nano-manufacturing processes, such as nano-indentation [17], nano-machining [18,19], and nano-forming [20]. Nevertheless, the MD simulation literature on material processing that considers material deformation under wet condition is scarce. We believe that the results from this work on nano-indentation can also shed light on its tribological effects for other nano-manufacturing processes.

The remainder of the paper is arranged as follows. The next section briefly explains the construction of MD simulation models and introduces the indentation process parameters for the simulation cases. Thereafter, the simulation results under dry indentation and wet indentation are compiled. They include the comparisons of load–displacement curves, calculated hardness and Young's modulus values, the distributions of friction and normal forces along the indenter/work interface, and stress distribution within the work material. Finally, conclusions are drawn in the final section.

## Methods

**Figure 1:**
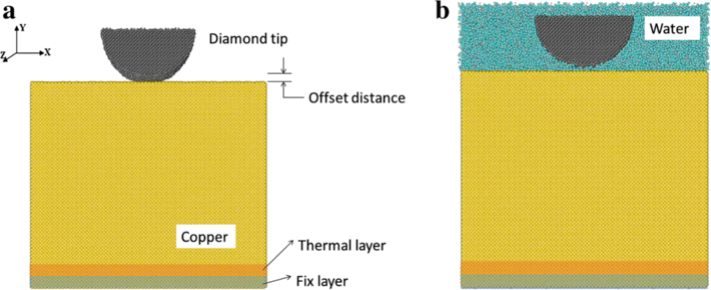
Schematic of MD simulation models for (a) dry nano-indentation and (b) wet nano-indentation.

Three-dimensional (3D) MD simulation models are constructed to study the indentation processes on single-crystal copper by a half-cylinder diamond indenter. For wet indentation, water molecules are added to fill the gap between the indenter and the work material in the system. For dry indentation, no water molecules are added. We employ LAMMPS, an open-source software developed by Sandia National Laboratory [21], to carry out the simulation computation. Figure 1 shows the schematic of MD simulation models for dry and wet indentations. The dimension of the copper work material is 247 × 216 × 70 Å^3^ (X, Y, and Z directions, respectively) for all simulation cases, and it consists of 306,000 copper atoms. The copper indentation surface is a (1 1 1) plane. The indenter has a radius of 50 Å, consisting of 46,000 carbon atoms. At the initial stage, the offset distance between the indenter and the work material is 5 Å. For wet indentation cases, the entire indenter is submerged in water, so both the indenter and the work material are in contact with water. In this case, 90,324 water atoms are contained in the system, including 60,216 hydrogen atoms and 30,108 oxygen atoms. Meanwhile, two special layers are defined in the copper work material, namely a thermal layer and a fixed layer. The fixed layer is located at the bottom of the work material, and it acts as a base to avoid any movement of the work material. The thermal layer is located right above the fixed layer, and it acts as a heat sink to maintain the temperature of the simulation system. In addition, since the simulation size is extremely small, a periodic boundary condition is applied along the Z direction so that the simulation box is replicated throughout the space to form an infinite lattice. This can effectively mitigate a spurious size effect when investigating the behavior of an isolated system.

In this study, we compare wet nano-indentation with dry nano-indentation by focusing on the tool/material interaction and process performances. Meanwhile, we consider the potential effect of indentation speed by including three levels of indentation speed. As a result, six simulation cases are created. Table 1 presents the detailed parameters of the six cases. Note that the indentation speeds are set high due to the high computational demand of MD simulation. For instance, it takes a cluster of 64 Intel i7 processor cores about 35 h to finish the computation of case 1.

**Table 1:** Nano-indentation parameters for the six simulation cases

**Case**	**Depth of indentation (Å)**	**Indentation speed (m/s)**	**Retraction speed (m/s)**	**Water molecules**
1	40	10	10	Yes
2	40	10	10	No
3	40	100	100	Yes
4	40	100	100	No
5	40	1	1	Yes
6	40	1	1	No

The simple point charge (SPC) liquid water model is adopted to describe the water molecules. In this model, one water molecule includes three centers of concentrated charge - a positive charge on two hydrogen atoms and an excess negative charge on one oxygen atom. The water molecules are modeled as a rigid isosceles triangle, and they interact via the Lennard-Jones (LJ) potential [22], in which the potential energy is calculated as

(1) $$V_{\mathrm{ij}}=4\epsilon \left[{\left(\frac{\sigma }{r}\right)}^{12}-{\left(\frac{\sigma }{r}\right)}^{6}\right]$$

where *σ* determines the distance at which the two particles are at equilibrium, *ϵ* is the strength of the interaction, and *r* is the distance between the particles. The parameters have different constant values for different interacting particles. The LJ potential is also applied to describe the Cu-O and the C-O potential energy for water-copper and water-carbon interactions, respectively. The values of *σ* and *ϵ* for Cu-H and C-H pairs on water-copper and water-carbon interactions are estimated via the Lorentz-Berthelot law [23]:

(2) $$\sigma_{\mathrm{ij}}=\frac{\sigma_{\mathrm{ii}}+\sigma_{\mathrm{jj}}}{2}$$

(3) $$\epsilon_{\mathrm{ij}}=\sqrt{\epsilon_{\mathrm{ii}}\epsilon_{\mathrm{jj}}}$$

The detailed parameters and values for all LJ interaction pairs are listed in Table 2.

**Table 2:** LJ potential parameters for O-O, O-Cu, O-C, C-H, and Cu-H atom pairs

**Parameter**	**O-O**	**O-Cu**	**O-C**	**C-H**	**Cu-H**
Equilibrium distance (*σ*, Å)	3.166	2.744	3.6	2.81	2.135
Cohesive energy (*ϵ*, 10^−3^ eV)	6.736	62.0	5.5	2.12	22.48
Cutoff distance (Å)	9.8	7.0	7.0	7.0	7.0
Bond length (Å)	1				
H-O-H angle (deg)	109.47				
*q*_O_	−0.847 *e*				
*q*_H_	(*q*_O_)/2				

The Cu-C interaction between the copper atoms in the work material and the carbon atoms in the indenter is calculated by the Morse potential [24,25], in which the energy is formulated as

(4) $$U=D\left\{\exp\left[-2\alpha \left(r_{\mathrm{ij}}-r_{0}\right)\right]-2\;\exp\left[-\alpha \left(r_{\mathrm{ij}}-r_{0}\right)\right]\right\}$$

where *α* is the elastic modulus and *r*_*ij*_ and *r*_0_ denote the actual distance and the equilibrium distance between paired atoms, respectively. The parameters for the Cu-C pair are summarized in Table 3.

**Table 3:** Morse potential parameters for the C-Cu pair interaction

**Parameter**	**Value**
Cutoff distance (Å)	6.5
Equilibrium distance *r*_0_ (Å)	2.22
Elastic modulus *α* (Å)	1.70
Cohesive energy *D* (eV)	−0.10

Within the copper work material, the interaction between copper atoms is described by the embedded atom method (EAM) potential, originally proposed by Daw and Baskes in 1984 [26]. The EAM potential is an approximation describing the energy between two atoms, and it is particularly appropriate for metallic systems. The total energy is given by

(5) $$E_{\mathrm{tot}}=\frac{1}{2}\sum_{i\neq j}V\left(r_{\mathrm{ij}}\right)+\sum_{i}F\left(\rho_{i}\right)$$

(6) $$\rho_{i}=\sum_{j}\phi \left(r_{\mathrm{ij}}\right)$$

The total energy is composed of the embedding energy *F*(*ρ*_*i*_) and the short-range pair potential energy *V*(*r*_*ij*_) between specific atoms *i* and *j*. *ρ*_*i*_ is the electron density contributed by other atoms at site *i*, and *φ*(*r*_*ij*_) is the electron density from the site of atom *j* to the site of atom *i*. Table 4 summarizes the detailed parameters and values of the EAM potential for the Cu-Cu interaction.

**Table 4:** **EAM potential parameters for the interaction among Cu atoms**[27]

**Parameter**	**Value**
Lattice constant	3.62 Å
Cohesive energy	−3.49 eV
Bulk modulus	137 GPa
*C*'	23.7 GPa
*C*_44_	73.1 GPa
Δ(Ebcc − Efcc)	42.7 meV
Δ(Ehcc − Efcc)	444.8 meV
Stacking fault energy	39.5 mJ/m^2^
Vacancy	1.21 eV

Indentation force is calculated by summing up the force acting on every carbon atom in the indenter, and the force of neighbor atoms of a specific atom is also summed:

(7) $$F=\sum_{i=1}^{N_{T}}\sum_{j}f_{\mathrm{ij}}$$

(8) $$f_{\mathrm{ij}}=\frac{\partial U\left(r_{\mathrm{ij}}\right)}{\partial r_{\mathrm{ij}}}$$

where *N*_*T*_ is the number of carbon atoms in the diamond indenter and *f*_*ij*_ is the individual interaction force from atom *j* acting on atom *i*.

Each of the stress components *S*_*xx*_, *S*_*yy*_, *S*_*zz*_, *S*_*xy*_, *S*_*xz*_, and *S*_*yz*_ of each atom is calculated during the indentation process. *χ* represents the virial stress component of each atom:

(9) $$\chi =\frac{1}{\Omega }\sum_{i}^{N}(m_{i}v_{i}⊗v_{i}+\frac{1}{2}\sum_{i\neq j}r_{\mathrm{ij}}⊗f_{\mathrm{ij}})$$

where Ω is the volume domain within the cutoff distance of atom *i*, *v*_*i*_ is the velocity of atom *i,* the sign ⊗ means the tensor product of vectors, and *N* is the total number of atoms in the domain. In addition, the equivalent stress can be calculated by following equation:

(10) $$\begin{array}{l} S=\\ \;\sqrt{3\left(S_{\mathrm{xy}}^{2}+S_{\mathrm{yz}}^{2}+S_{\mathrm{xz}}^{2}\right)+\frac{1}{2}\left[{\left(S_{\mathrm{xx}}-S_{\mathrm{yy}}\right)}^{2}+{\left(S_{\mathrm{xx}}-S_{\mathrm{zz}}\right)}^{2}+{\left(S_{\mathrm{yy}}-S_{\mathrm{zz}}\right)}^{2}\right]} \end{array}$$

## Results and discussion

### Indentation morphology and force

**Figure 2:**
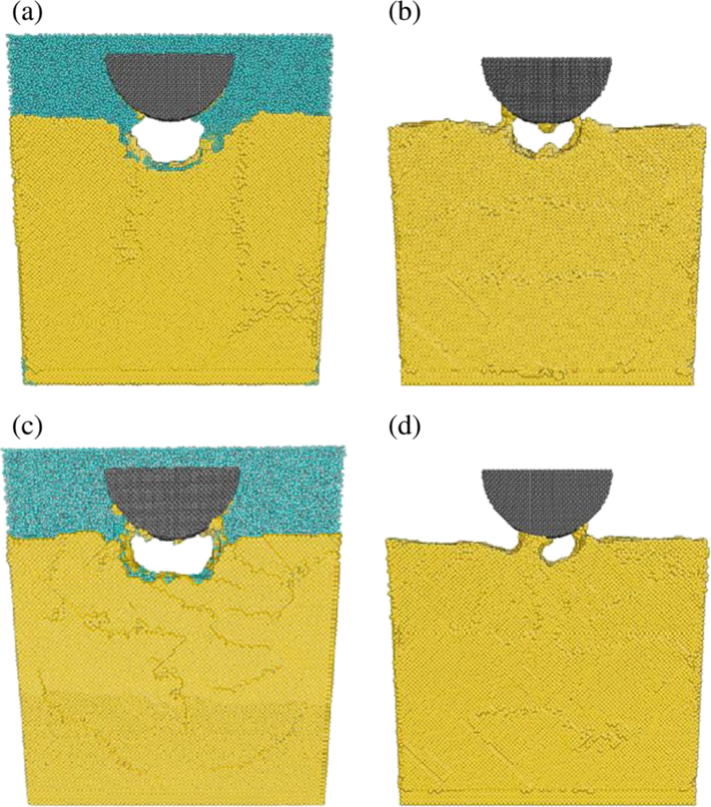
Indentation morphologies for (a) case 1, (b) case 2, (c) case 3, and (d) case 4.

The indentation morphology after the indenter is fully retracted is shown in Figure 2. The comparison can be established between cases 1 and 2 at 10 m/s of indentation speed, as well as cases 3 and 4 at 100 m/s of indentation speed. It can be seen that for each comparison pair, the existence of water reduces the sticking of copper atoms on the indenter surface. Also, there are water molecules remaining in the indentation area for wet indentation cases. For both indentation speeds, the indentation depth under wet condition is clearly deeper than that under dry condition. The result indicates that the addition of water molecules helps preserve the indentation geometry during tool retraction by reducing the atom adhesion effect between the indenter and the work piece. This finding might be of interest for the tool-based ultra-precision manufacturing, where tight control of deformation geometry is often called for.

**Figure 3:**
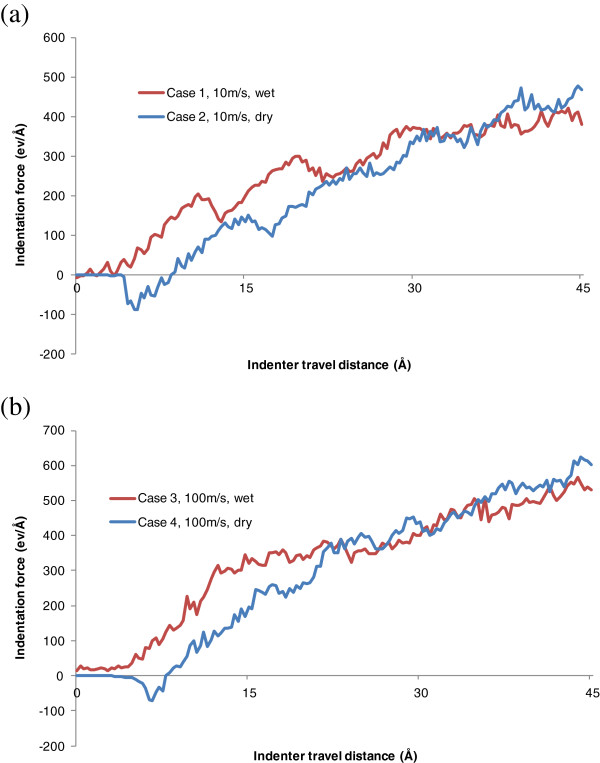
Effect of water molecules on indentation force at the speeds of (a) 10 and (b) 100 m/s.

As shown in Figure 3, the evolutions of indentation force with respect to tool penetration distance under wet and dry indentations are compared for the two indentation speeds of 10 and 100 m/s, respectively. During the initial period of dry indentation, the curves start with zero indentation force, which indicates that the distance between the copper surface and the indenter is larger than the cutoff distance for any meaningful atomic interaction. After that, the indentation force becomes negative, which implies that the attraction effect between the indenter and the copper work material overcomes the repulsion effect. As the indenter further advances, the indentation force then alters to repulsive and increases with the progress of indentation. For wet indentation cases, the existence of water molecules between the indenter and the work material generates repulsive force at the beginning. The force is large enough to overcome the combined attraction force on the indenter, so the indentation force seldom appears to be negative. Besides, the repulsive force between the indenter and the water results in higher indentation force when the indentation depth is less than 2 nm.

**Figure 4:**
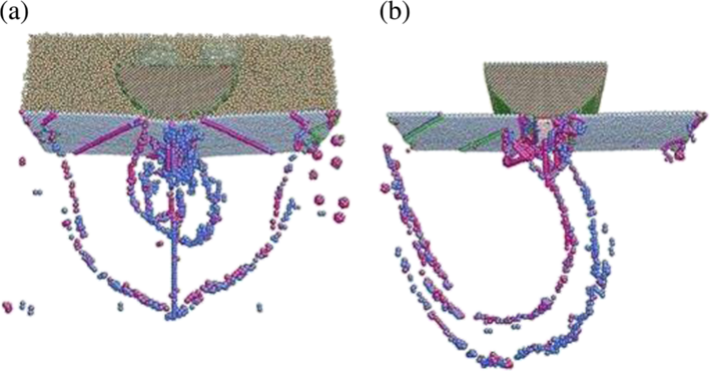
Dislocations in the work material at 8-Å indentation depth for (a) case 1 and (b) case 2.

Fluctuation can be observed in all curves. This is introduced by complex dislocation movement of atomic layers in the single-crystal copper during the indentation process. Similar observations are reported by other studies as well [28,29]. Higher indentation force should be linked to more drastic copper atom dislocation movement and entanglement. This can be confirmed by the dislocation movements of cases 1 and 2, as shown in Figure 4. For both cases, when the indenter penetrates into the surface of the copper material, the dislocation embryos immediately develop from the vacancies in the vicinity of the indenter tip. Compared with those in dry indentation (case 2), the dislocation embryos beneath the indenter in wet indentation (case 1) are larger, and the atomic glides on the surface are more drastic as well. However, both cases seem to have the same glide direction, which is along the slip vectors associated with the FCC (111) surface. The more drastic dislocation movement as seen in wet indentation is clearly contributed to the higher indentation force caused by the repulsive force between the indenter and the water molecules.

However, for both 10 and 100 m/s speeds, the indentation force for dry indentation starts to overtake that for wet indentation when the indentation depth reaches 3.3 nm. This phenomenon can be attributed to the change of friction force between the indenter and the work material due to the addition of water. When the indentation depth is less than a critical value, the resultant reduction of indentation force is too small to compensate the resistant force of water molecules between the indenter and the work material. When the indentation depth is beyond the critical value, the beneficial tribological effect is sufficient to compensate the resistant force. As a result, the indentation force in the late stage for wet indentation is smaller than that for dry indentation.

**Figure 5:**
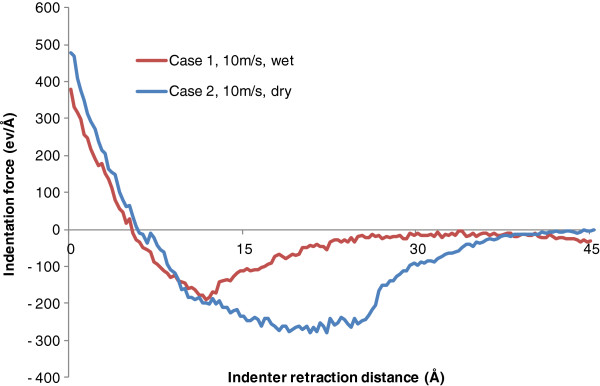
Effect of water molecules on indentation force during tool retraction.

In addition, Figure 5 illustrates the effect of water on indentation force during the tool retraction process by comparing cases 1 and 2. For both wet and dry indentations, the indentation force decreases quickly at the beginning and reaches the equilibrium state at the retraction distance of about 0.7 nm. For dry indentation, the peak adhesion force is 270 eV/Å which occurs at the retraction distance of 2.1 nm. For wet indentation, the indenter/work adhesion is considerably reduced. The peak adhesion force is 205 eV/Å which occurs at the retraction distance of 1.1 nm. The adhesion region is also much narrower in wet indentation. In addition, for both curves, the indentation force gradually reduces to zero as the indenter is withdrawn to its original position. In summary, the existence of water can significantly reduce the attraction effect between carbon atoms and copper atoms, and the magnitude of the overall attraction force on the indenter decreases by 30.1%. This can be reflected by the final indentation morphology comparison made in Figure 2.

### Hardness and Young's modulus

Based on the indentation load *P* and the measured actual projected contact area *A*_c_, the hardness of the work material can be calculated as

(11) $$H=\frac{P}{A_{c}}.$$

**Figure 6:**
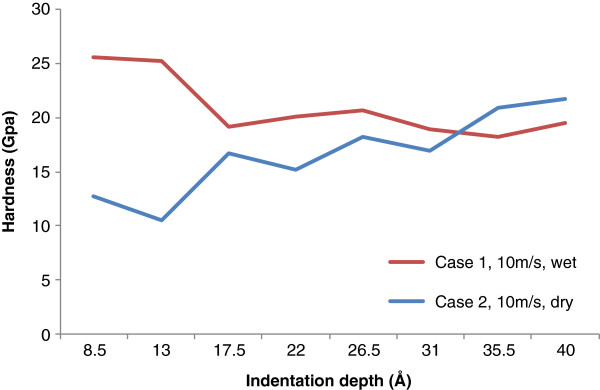
Hardness value with respect to indentation depth under dry and wet conditions.

In this way, the evolution of hardness with the penetration depth of the indenter for cases 1 and 2 is obtained, as shown in Figure 6. For wet indentation, the maximum hardness is observed at the beginning of the indentation process and gradually decreases to a stable value of about 19.4 GPa. The high hardness value at the beginning of wet indentation can certainly be attributed to the high repulsion effects between the water and the tool, as well as between the water and the work material. By contrast, in dry indentation the hardness value overall increases with the progress of indenter engagement. At the maximum engaging depth, the calculated hardness value is about 22.0 GPa, which is significantly higher than that of dry indentation. Similar to the trend of indentation force, the calculated hardness value for dry indentation starts to overtake wet indentation at the indentation depth of about 3.3 nm.

The hardness curve for wet indentation demonstrates the ISE, which means that the calculated hardness decreases with the increase of loading/penetration. On the other hand, the hardness-depth curve for dry indentation exhibits the reverse ISE, which means that the hardness increases with the increase of loading/penetration. These findings are not very common for numerical studies in the literature, but they are fairly consistent with experimental studies in the literature at larger scales. For instance, the reverse ISE in dry indentation is reported in several studies [30-32], and the regular ISE in lubricated indentation is also reported [14-16]. In particular, the reverse ISE phenomenon has not been fully understood. Speculated reasons include the existence of a distorted zone near the crystal-medium interface [33], the applied energy loss due to specimen chipping around the indentation [34], and the generation of median or radial cracks during indenter loading half-cycle [30].

It is commonly accepted that the loading part of the indentation process is considered an elastic–plastic response, while the unloading part is a pure elastic rebound of the material and it is only related to the elastic property of the material. Loubet et al. [35] proposed a flat-ended punch model to estimate the stiffness of the specimen. Later, Hay et al. [36] showed that since the boundary conditions used in elastic contact models allow for inward displacement of the surface, a shape factor of the indenter, *β*, is introduced:

(12) $$S=\frac{dP}{dh_{s}}=2\beta E_{r}\sqrt{\frac{A}{\pi }}$$

where *S* is the stiffness of the test material, obtained from the initial unloading slope at maximum load and maximum depth; *A* is the projected contact area of the indenter at maximum loading condition; and *E*_r_ is the reduced modulus or combined modulus. The value of shape factor *β* for a cylindrical indenter is 1 [37]. *E*_r_ represents a balance between Young's modulus of the sample, *E*_s_, and that of the indenter, *E*_i_, because both the sample and the indenter experience elastic deformation during the indentation process:

(13) $$\frac{1}{E_{r}}=\frac{1-v^{2}}{E}+\frac{1-v_{0}^{2}}{E_{0}}$$

where *E* and *v* are Young's modulus and Poisson's ratio for the specimen, respectively, and *E*_0_ and *v*_0_ are the same parameters for the diamond indenter, respectively. The copper property used in this study's calculation is *v* = 0.3 [38]. Since the diamond indenter in this study is assumed to be perfectly rigid with *E*_0_ = ∞, Equation 13 can be simplified as

(14) $$E_{r}=\frac{E}{1-v^{2}}.$$

Combining it with Equation 12, we obtain

(15) $$E=\frac{1-v^{2}}{2\beta }\sqrt{\frac{\pi }{A}}\frac{dP}{dh_{s}}.$$

**Figure 7:**
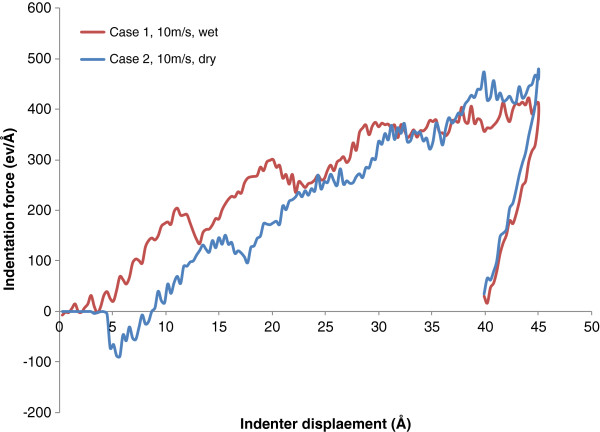
Load-unload curve for wet and dry indentations (cases 1 and 2).

In the end, the calculated Young's modulus values of copper are 194.1 and 255.3 GPa for wet indentation (case 1) and dry indentation (case 2), respectively. Young's modulus measured by dry indentation is significantly greater than that measured by wet indentation. This is attributed to its higher stiffness as observed during the initial unloading period from the load-unload curve, as shown in Figure 7.

Furthermore, regarding the hardness and Young's modulus measurements of the copper material, a comparison between this study and the literature is made in Table 5. The results of MD simulation in this study are compared with the results obtained in other MD simulation studies of dry nano-indentation, as well as the experimental measurements obtained at micro- and nano-scale in the literature. From the table, the hardness and Young's modulus values obtained in our study are overall consistent with other MD simulation studies in the literature. However, all the MD simulation studies produce higher values of hardness and Young's modulus than the existing experiment studies. The large discrepancy is due to the scale differences between MD simulation and experiment. The simulation assumes a perfect structure of single-crystalline copper lattice at the nano/atomistic scale, which is smaller than any existing nano-indentation experiments. Within the regular high-purity copper, many defects exist such as grain boundaries and precipitates at the grain boundaries. These defects significantly affect the deformation behavior of the material and weaken the work material.

**Table 5:** Comparison of MD simulation results with the literature

	**Hardness (GPa)**	**Young**'**s modulus (GPa)**
Case 1 of this study - wet indentation	19.5 to 25.5	194.1
Case 2 of this study - dry indentation	12.7 to 21.7	255.3
MD simulation by Fang et al. [37]	20.4 to 43.4	283.4 to 444.9
MD simulation by Leng et al. [38]	23	N/A
Nano-indentation experiment [36]	7.1 to 10	135
Micro-indentation experiment	2.1 [39]	116 to 126 [40]

Note that the mechanisms of dislocation development with the presence of imperfections and grain boundaries in nano-indentation processes are investigated by numerical approaches in the literature. In this regard, the representative studies cover the typical research topics of dislocation nucleation and defect interactions [41], vacancy formation and migration energy, interstitial formation energy, stacking fault energy [42], coherent twin boundaries and dislocations [43], and the effect of grain boundary on dislocation nucleation and intergranular sliding [44]. In addition, Shi and Verma [27] compared the nano-machining processes of a monocrystalline copper and a polycrystalline copper by MD simulation. The results indicate that the presence of grain boundaries significantly reduces the cutting force and stress accumulation inside the workpiece by up to 40%. However, the focuses of these studies are not about the calculation of hardness and Young's modulus, and certainly they do not tackle the tribological effects of any liquid. As such, it will be interesting to carry out such investigation on nano-indentation simulation of polycrystalline structures in the near future.

### Friction along the tool/work interface

**Figure 8:**
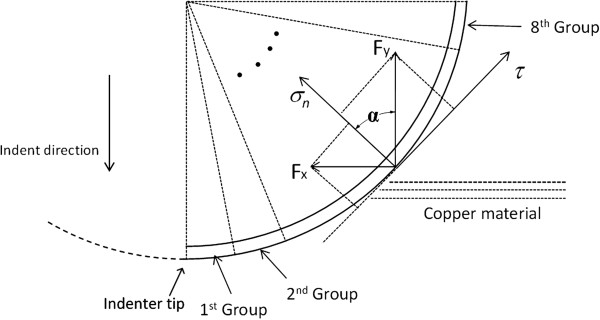
Atom grouping for friction analysis along the indenter/work interface.

To investigate the tribological effect of water molecules in nano-indentation, the normal force and friction force distributions along the indenter/work material interface are obtained. As shown in Figure 8, a thin surface layer of the indenter is considered, and the atoms in this layer are evenly divided into eight groups. Each group contains about 450 carbon atoms, and the force acting on each atom group is individually computed. Note that each group is identical, so the groups have the same contact area. As such, the force distributions along the indenter/work material interface are actually equivalent to the stress distributions.

The friction force *τ* and the normal force *σ*_*n*_ acting on each group are calculated by the following equations:

(16) $$\sigma_{n}=F_{y}\cos\alpha +F_{x}\sin\alpha$$

(17) $$\tau =F_{y}\sin\alpha +F_{x}\cos\alpha$$

where *F*_*x*_ and *F*_*y*_ are the average horizontal and vertical force components of each group, respectively.

**Figure 9:**
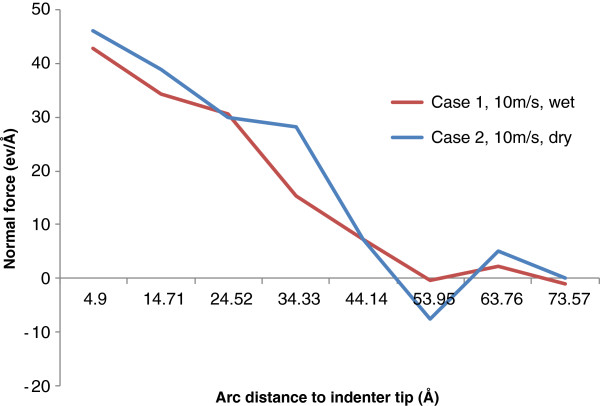
Normal force distribution along the indenter/work interface.

The distributions of normal force on the indenter/work interface at the maximum penetration position for cases 1 and 2 are shown in Figure 9. The two curves exhibit similar downward trends with the increase of ‘arc distance to the indenter tip’. The maximum normal force occurs at the very beginning of the curves, which indicates the group closest to the indenter tip. Along the interface, the normal force gradually decreases to zero at about 5 nm to the indenter tip and no obvious normal force can be observed beyond this distance. By comparison, the normal force on the interface for wet indentation is overall slightly smaller than that for dry indentation.

**Figure 10:**
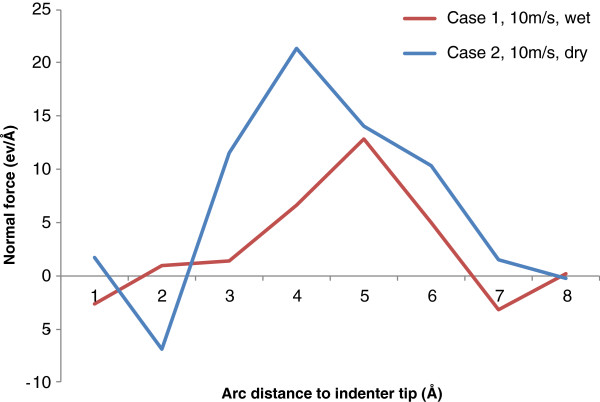
Friction force distribution along the indenter/work interface.

Figure 10 presents the distributions of friction force along the indenter/work interface for cases 1 and 2. For both cases, the friction force in the vicinity of the indenter tip is small, but it increases rapidly as the distance to the indenter tip increases. For dry indentation (case 2), the maximum friction force occurs at about 3.4 nm to the indenter tip, and the value is 21 eV/Å. For wet indentation, the maximum friction force on the interface is 12.8 eV/Å, and it is obtained at 4.4 nm to the indenter tip. This represents a reduction of 39% in terms of the maximum friction force. Also, for both cases, after the maximum friction force is reached, friction force gradually reduces to zero as the distance to the indenter tip increases. By comparing the two curves, it can be seen that the existence of water can significantly reduce the friction force along the indenter/work interface. This represents a major beneficial tribological effect. The reduction of friction force on the interface is believed to result in smaller indentation forces and a smaller hardness value at maximum indentation depth. This is supported by the micro-hardness testing results reported by Li et al. [16], whose study confirms that the indenter/specimen interfacial friction has a significant effect on the low-test-load indentation micro-hardness based on the traditional power law and proportional specimen resistance model.

**Figure 11:**
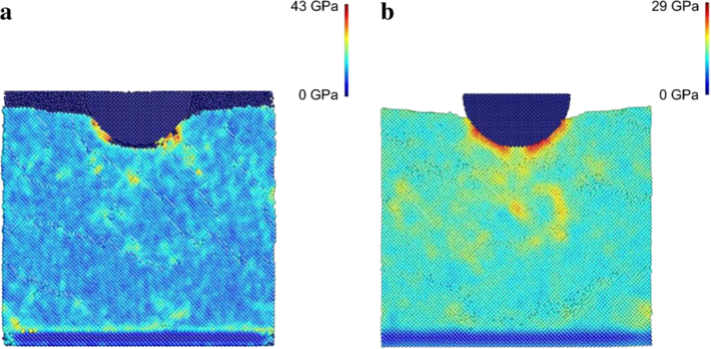
Equivalent stress distribution in nano-indentation for (a) case 1 and (b) case 2.

Besides, the equivalent stress distributions of nano-indentation are obtained for cases 1 and 2. As shown in Figure 11, the stress gradient in case 1 is steeper than that in case 2. The maximum equivalent stress is 43 GPa for wet indentation, which is located along the indenter/work interface and approximately consistent with the peak friction force location in Figure 10. Meanwhile, the maximum equivalent stress is 29 GPa for dry indentation, which has a similar location.

### Influence of indentation speed

**Figure 12:**
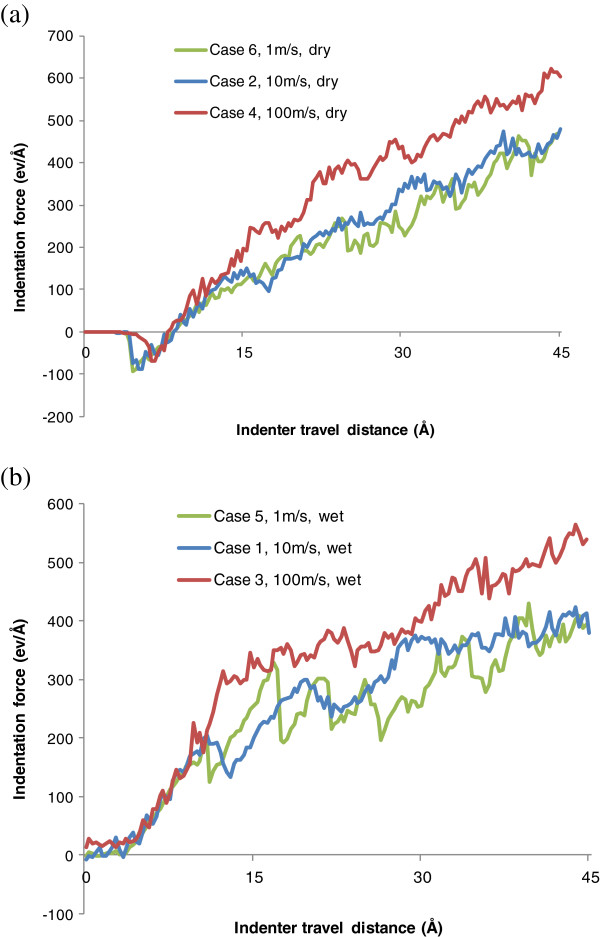
**Effect of indentation speed on indentation force evolution. (a)** Dry condition for cases 6, 2, and 4. **(b)** Wet condition for cases 5, 1, and 3.

The influence of indentation speed is also examined. Here, we group cases 1, 3, and 5 to discuss the influence of indentation speed in wet indentation and cases 2, 4, and 6 for dry indentation. Two general observations can be obtained. First of all, the indentation force evolutions are compared, as shown in Figure 12. It can be seen that for both dry and wet nano-indentations, the indentation speed of 100 m/s generates the highest overall indentation force. The force increase is pronounced when the speed increases from 10 to 100 m/s. However, the force increase is not significant when the speed changes from 1 to 10 m/s. Second, within the range of the indenter travel distance of 10 Å, the three curves under dry or wet indentation overlap each other and the indentation force almost linearly increases with the travel distance. As the indenter tip further advances, the three curves start to deviate from each other.

**Figure 13:**
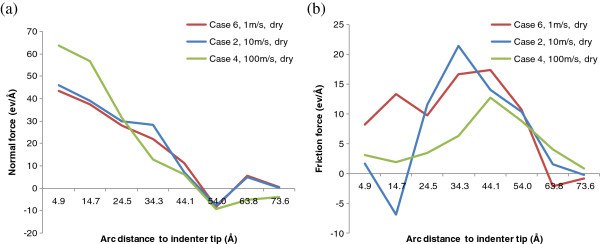
Indentation speed effect on (a) normal and (b) friction force distributions under dry indentation.

Moreover, we also analyze how the indentation speed affects friction behaviors along the indenter/work interface. Figure 13 shows the normal and friction force distributions under dry condition for cases 6, 2, and 4. It can be seen that under dry indentation, the normal force of case 4 (100 m/s speed) is significantly higher than those of cases 6 and 2 (1 and 10 m/s, respectively) at surface locations close to the indenter tip. The difference diminishes at the position about 2.5 nm to the indenter tip, in which all three indentation speeds have approximately the same normal force. When the surface position to the indenter tip further increases, the normal force at 100 m/s becomes smaller than those at 1 and 10 m/s, and the 1 m/s curve is overall slightly lower than the 10 m/s curve in terms of normal force. The trend in normal force is consistent with that observed in indentation force comparison, as shown in Figure 12a. In terms of friction force distributions, the three curves have a similar shape, and the peak friction force is located around 3.4 to 4.4 nm to the indenter tip depending on the indentation speed. Also, the overall (total) friction force decreases with the increase of indentation speed.

**Figure 14:**
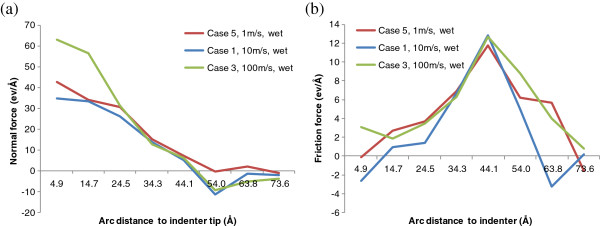
Indentation speed effect on (a) normal and (b) friction force distributions under wet indentation.

In the mean time, Figure 14 compares the normal and friction distributions under wet indentation at the indentation speeds of 1 m/s (case 5), 10 m/s (case 1), and 100 m/s (case 3). Compared with Figure 13a, similar observations can be made among the three normal force curves under wet indentation. Also, the friction force curves in Figure 14b have fairly consistent shapes, and the peak friction force is always located at around 4.4 nm to the indenter tip.

## Conclusions

This research investigates nano-indentation processes with the existence of water molecules by using the numerical approach of MD simulation. The potential tribological benefits of water or other liquids, as well as the influence on material property measurements, are intriguing to nano-indentation. This also applies to other tool-based precision manufacturing processes. By configuring 3D indentation of single-crystal copper with a diamond indenter, six simulation cases are developed. Based on the results, the major findings can be summarized as follows:

•Compared with dry indentation, wet indentation incurs higher indentation force during the initial penetration of the indenter, but lower force during the full penetration period.

•Wet indentation can effectively reduce the adhesion between the atoms of the work material and the atoms of the indenter. It helps preserve the final indentation shape and geometry after the indenter is retracted.

•In dry indentation, the hardness-indentation depth curve exhibits the reverse indentation size effect. In wet indentation, the curve exhibits the regular indentation size effect.

•By analyzing the force distributions along the indenter/work interface, it is found that the existence of water molecules can significantly reduce the friction force, but not the normal force.

•In dry indentation, the maximum indentation force increases from 468.0 to 549.7 eV/Å as the indentation speed increases from 10 to 100 m/s. In wet indentation, the maximum indentation force increases from 423.2 to 565.6 eV/Å with the same increase of speed. However, the increase of indentation force is much less significant when the speed increases from 1 to 10 m/s.

## Competing interests

Both authors declare that they have no competing interests.

## Authors’ contributions

Mr. YW carried out the molecular dynamics simulation. Dr. JS conceived of the study and developed the simulation model. Both authors analyzed the results and drafted the manuscript. Both authors read and approved the final manuscript.
